# Decoupling of the Growing Exports in Foreign Trade from the Declining Gross Exports of Embodied Energy

**DOI:** 10.3390/ijerph19159625

**Published:** 2022-08-05

**Authors:** Wenmei Kang, Mou Wang, Ying Chen, Ying Zhang

**Affiliations:** 1 Faculty of Applied Economics, University of Chinese Academy of Social Sciences, Beijing 102488, China; 2 Research Center for Sustainable Development, Chinese Academy of Social Sciences, Beijing 100732, China; 3 Research Institute for Eco-civilization, Chinese Academy of Social Sciences, Beijing 100710, China

**Keywords:** decoupling, economic growth, foreign trade, embodied energy, global climate governance

## Abstract

Transforming the growth mode and realizing green and low-carbon development has been a global consensus and an important governance concept of socialism with Chinese characteristics in the new era. Whether economic growth can be decoupled from carbon emissions and energy consumption is a key indicator for measuring green and low-carbon development and is an inevitable requirement for achieving the goal of carbon neutrality before 2060. Based on the input–output tables for 2002, 2007, 2012, and 2018, this article calculated the embodied energy of export in China’s foreign trade and studied the elastic relationship and trend between the growth of foreign trade exports and the total embodied energy of export since China’s accession to the WTO. The following conclusions were drawn: (i) The embodied energy of China’s export was strongly decoupled from total export for the first time from 2012 to 2018, signaling that China’s economic, industrial, and energy structures entered a new stage. It was also the first strong decoupling achieved in the process of decoupling economic growth from energy consumption for the adoption of a low-carbon development path. Due to the pressure of international competition, the export sector had a relatively advanced level of efficiency, so it achieved decoupling earlier than the overall manufacturing sector and the consumption sector, which was in line with economic laws and the characteristics of China’s development stage. (ii) From 2007 to 2018, the embodied energy of export occupied a much smaller proportion of China’s total energy consumption, falling from the peak of 31.48% in 2007 to 26.57%, a drop of 4.91 percentage points. It showed that a larger share of energy consumption had taken place domestically and that the model mainly relying on export expansion to drive economic growth had begun to adjust. The conclusion of this research could also support the assertion of ‘accelerating the construction of a new development pattern with the domestic economic cycle as the main body and the domestic and international dual cycles promoting each other’ from the perspective of external exports and energy consumption. (iii) A causal analysis of the decoupling between the embodied energy of export and export volume demonstrated that, from 2002 to 2007 and from 2007 to 2012, the embodied energy of export and total export maintained the same direction but had different growth rates. The increase in total export volume was the main reason affecting the embodied energy of export. With the rapid growth of total export volume, the embodied energy of export was also growing rapidly. From 2012 to 2018, the embodied energy of export declined, and an analysis showed that the ‘total energy consumption coefficient’, i.e., technology effect, was the primary cause of the decline. With China’s high-quality development, green transformation, and other strategic advancements, the decoupling trend is expected to continue and expand to a larger economic field.

## 1. Introduction

Building an ecological civilization, staying true to the new development philosophy, and being committed to the harmony between man and nature, these are not only the ideas advocated by China, but also the core elements of the United Nations’ 2030 Sustainable Development Goals. Green and low-carbon development is an inevitable way to build an ecological civilization and realize sustainable development featuring harmony between man and nature. It is also a necessary condition required for achieving carbon neutrality before 2060. Since the 1990s, when global governance was carried out by human society to address climate change, 21 countries, including Germany, the United Kingdom, and the United States, have successively decoupled economic growth from carbon emissions between the year 2000 and 2014 [1]. These countries’ fossil energy consumption and carbon emissions followed the laws of the environmental Kuznets curve and began to slowly decline after reaching the peak, while their economic aggregate maintained a growth trend, which can be seen as the manifestations of absolute decoupling or strong decoupling. China is still in a period of rapid economic and social development, and its development stage is still on the left wing of the environmental Kuznets curve. To achieve absolute decoupling of economic development from energy consumption and carbon emissions, the country must first go through a stage in which energy consumption gradually reaches its peak. After some key factors affecting energy consumption and carbon emissions gradually come into play and reach their peaks, it is then possible to gradually achieve the absolute decoupling of China’s economic development from energy consumption and carbon emissions. These key factors can be decomposed into energy consumption and carbon emissions in manufacturing, household consumption, infrastructure construction, and export. The energy consumption of these sectors or fields will not reach peaks at the same time. According to the development experience of other countries, the production sector generally reaches its peak before the consumption sector [2]. Due to pressure from international competition, the export sector is more advanced in efficiency and may reach the peaks of energy consumption and carbon emissions sooner than the entire manufacturing industry. Studying the peaking time and trends of these key factors is the basis for understanding and grasping the trends of China’s energy consumption and carbon emissions, as well as the basis for adjusting and formulating China’s policies relating to industrial production, foreign trade, and energy consumption.

As for decoupling, an economic phenomenon, researchers study the elastic relationship of relative changes in two variables to reflect the development and evolution trends of different variables and use the decoupling elasticity coefficient or the decoupling index to show the degree and direction of evolution trends. The decoupling index model, proposed by Tapio [3] when studying the relationship between traffic capacity and GDP, is divided into eight categories, including weak decoupling and strong decoupling, according to the values and signs of decoupling indexes. Diakoulaki et al. [4], Pan et al. [5], and Sheng et al. [6] used the decoupling index to study the relationship between economic growth and carbon emissions in China and other countries. Zhang et al. [7] defined and studied the different nature and stages of decoupling and expanded the decoupling index model into ten categories based on research by Tapio [3]. Many expressions of decoupling can be classified into two categories from the perspective of development: (i) economic development and the growing trend of energy consumption or carbon emissions change in the same direction but in different ranges, which can be called relative decoupling or weak decoupling, a category which in some studies is subdivided into concepts such as strong relative decoupling and weak relative decoupling; (ii) economic development and the growing trend of energy consumption or carbon emission change in the opposite directions, i.e., increment versus decrement in any ranges, which can be called absolute decoupling or strong decoupling. The relative decoupling between economic development and carbon emissions has been achieved in China, on which some studies have been carried out. However, China’s absolute decoupling from carbon emissions in all economic fields, such as production, consumption, and trade, has not yet been revealed. By studying the change trends and absolute decoupling phenomenon of China’s total value of export and embodied energy of export, this paper intends to reflect the characteristics of China’s current development in economy, trade, and production technology in order to provide a reference for the formulation of policies related to the socio-economic transition to green development, adjustments to industrial and energy structures, as well as participation in global climate governance.

With economic transformation and development and environmental pollution becoming the focus of attention, the rapid development of international trade has also profoundly shaped the global trade landscape, determining energy flow, industrial division, and cooperation modes in different regions while also influencing countries’ economic and industrial development trends and policies. In terms of the energy embodied in global trade, scholars across the world have conducted a lot of research on the embodied energy flow of trade commodities. Some of them revealed the patterns of embodied energy flowing with global trade from the aspect of the embodied energy flow in international trade. For example, Chen et al. [8] analyzed the embodied energy of the world’s economy and trade in the context of globalization in 2004 based on an input–output model, indicating that the United States is the world’s largest importer of embodied energy, while China is the world’s largest exporter of embodied energy during the same period; Mi et al. [9] used a multi-regional input–output (MRIO) table to analyze embodied carbon in the trade of major countries in the world from 2007 to 2012 and found that the total embodied carbon of China’s exports declined during the study period, though China was still a net exporter of carbon; Cui et al. [10] used the input–output model and found that, in 2007, the United States, Japan, the United Kingdom, Italy, and other countries were net importers of embodied energy while China, Russia, India, and other countries were net exporters of embodied energy; Guo et al. [11] found that from 1995 to 2014, the United States, Japan, France, Germany, and Brazil were net importers of embodied energy while Russia, South Korea, India, China, and Canada were net exporters of embodied energy. Some of them studied the characteristics and conditions of the embodied energy flow from the aspect of the embodied energy flow in particular countries. For example, Chen et al. [12] used the input–output model to calculate the embodied energy of China’s foreign trade from 2002 to 2006, with the results showing that China was a net exporter of embodied energy and that the embodied energy of export accounted for 20.7% of the country’s energy consumption that year. Qi et al. [13] used the input–output method to estimate the embodied carbon of China’s import and export from 1997 to 2006, with the results showing that China was a net exporter of embodied carbon during the study period. Tang et al. [14] studied embodied energy in the international trade of the United Kingdom as a developed country from 1997 to 2011 and inferred from the analysis that the United Kingdom was a net importer of embodied energy during the study period. Xu et al. [15] used the input–output method to analyze embodied carbon and carbon transfer emissions, with the results showing that China had been a net exporter of carbon in the Sino-British trade from 2004 to 2009. The results of studies by Yang et al. [16] and Wei et al. [17] showed that China had always been a net exporter of embodied energy in Sino-US trade. Chen et al. [18] and Xie et al. [19] analyzed the embodied energy of import and export trade based on the input–output tables published by the Organization for Economic Co-operation and Development (OECD) and conducted structural analyses on the embodied energy of export by using the structural decomposition analysis (SDA) model, from which they found that China was a net exporter of embodied energy from 1995 to 2005 and that the expansion of export scale was the main reason for the increasing embodied energy of export. Zhang et al. [20] calculated China’s embodied energy in trade from 1997 to 2013 at the levels of the country and specific industries based on an improved method, with the results showing that China was still a net exporter of embodied energy during the study period and that the net values of embodied energy in every Chinese sector comprise both surplus and deficit, with the surplus being dominant on the whole.

In general, the methodology of estimating the embodied energy of export needs to take into account two key issues: (i) The choice between the input–output method and life cycle assessment. Life cycle assessment is used to study embodied energy in the whole life cycle of commodities from a micro perspective in order to evaluate its impact on the environment and the pattern of embodied energy flows related to international trade. Due to the complexity and variety of import and export commodities, it is relatively difficult to use this method to obtain data and to accurately define the boundaries of energy consumption in each link, thus easily leading to repeated accounting or omissions. Mainly based on international and/or national input–output tables, the input–output method can more comprehensively reflect the participation of different countries and different sectors in international trade and more accurately measure the corresponding embodied energy flows. Therefore, most researchers choose the input–output method to carry out studies. (ii) Whether or not to deduct and how to deduct the imported intermediate inputs when calculating the embodied energy of export. The embodied energy of export refers to the total energy consumed in the production, manufacturing, logistics, and other processes of a country’s export, but does not include the energy consumed by imported intermediate inputs in the aforementioned processes. When deducting the energy consumed by imported intermediate inputs in the calculation of embodied energy of export, because the data in China’s competitive input–output tables do not include a list of imported intermediate input data, studies by Gu et al. [21], Liu et al. [22], and Zhang et al. [20] used the method of distribution in equal proportions to technically process imported intermediate inputs and worked out corresponding embodied energy of export. However, Pang et al. [23] did not deduct the embodied energy consumed by imported intermediate inputs when calculating the embodied energy of China’s exports and the results were higher than those calculated by Liu et al. [22].

Although there has been a range of studies conducted on China’s embodied energy in international trade, most of these studies are conducted in particular years, or based on input–output tables of particular years, reflecting the static situation or pattern of the international flow of embodied energy in the current period of input–output tables, thus certainly making them unable to reveal the changing trend of embodied energy of export that may be caused by China’s economic development and changes in the industrial and energy structure. However, understanding the changing trend can provide key information for formulating and adjusting the policies for future economic, industrial, and energy development. Since China acceded to the WTO in 2001, exports in foreign trade have become one of the main driving forces for rapid economic growth. The growth curves and characteristics of exports in foreign trade before and after China’s accession to the WTO can also be differentiated into two stages. Therefore, this paper focuses on the changing trend of embodied energy of export since China’s accession to the WTO. Based on the input–output tables (The extended input–output tables for 2005, 2010 and 2015 are also analyzed, but the results are for reference only because they are not actual survey tables) for 2002, 2007, 2012, and 2018 in China, this paper studies the trends of embodied energy of export through a relatively long time series based on the latest data, and by analyzing the trends of two variables, i.e., export scale and embodied energy of export, judges the elastic relationship of relative changes in the two variables and defines decoupling status and the degree and nature of the two variables. On that basis, the SDA weighted average method is used to decompose the factors affecting the changes in embodied energy of export from 2002 to 2018 and to explore the main reasons that lead to such changes.

## 2. Theoretical Models

The input–output (IO) method is an analysis method explored and created by Leontief in 1936 and has been widely used in the calculation of embodied energy in commodities for domestic and foreign trade [12,14,20]. The input–output model constructed in this paper for calculating the embodied energy of China’s export is as follows:

Suppose that China trades with *r* partners, among which each country or region owns *m* sectors. For country *p*, according to the horizontal quantitative relationships of the input–output tables, Equation (1) can be obtained.

(1) $$\sum_{j=1}^{m}a_{ij}^{p}*x_{j}^{p}+y_{i}^{p}=x_{i}^{p}(i=1,2,\ldots ,m)$$

where $a_{ij}^{p}$ is the direct consumption coefficient of sector No. *i* and $a_{ij}^{p}$ = $\frac{x_{ij}^{p}}{x_{j}^{p}}$, thus the direct consumption coefficient matrix is $A^{P}$,

$$A^{p}=\left[\begin{array}{cccccc} a_{11}^{p} & a_{12}^{p} & \cdots & a_{1j}^{p} & \cdots & a_{1m}^{p}\\ a_{21}^{p} & a_{22}^{p} & \cdots & a_{2j}^{p} & \cdots & a_{2m}^{p}\\ \vdots & \vdots & \vdots & \vdots & \vdots & \vdots \\ a_{i1}^{p} & a_{i2}^{p} & \cdots & a_{ij}^{p} & \cdots & a_{im}^{p}\\ \vdots & \vdots & \vdots & \vdots & \vdots & \vdots \\ a_{m1}^{p} & a_{m2}^{p} & \cdots & a_{mj}^{p} & \cdots & a_{mm}^{p} \end{array}\right]$$

$x_{j}^{p}$ is the total input of sector No. *j*, $y_{i}^{p}$ is the final demand of sector No. *i*, and $x_{i}^{p}$ is the total output of sector No. *i*, and when i=j, $x_{j}^{p}$ = $x_{i}^{p}$.

If $X^{p}={\left(x_{1}^{p},x_{2}^{p},\ldots ,x_{m}^{p}\right)}^{T}$, $Y^{P}={\left(y_{1}^{p},y_{2}^{p},\ldots ,y_{m}^{p}\right)}^{T}$, then the matrix relationship of Equation (1) can be expressed as:

(2) $$A^{P}*X^{P}+Y^{P}=X^{P}$$

By transposing the terms of Equation (2), we obtain:

(3) $$X^{P}={\left(I-A^{P}\right)}^{-1}Y^{P}$$

where ${\left(I-A^{P}\right)}^{-1}$ is the complete demand coefficient, defining that $B^{P}$ = ${\left(I-A^{P}\right)}^{-1}$. Thus, the element $b_{ij}^{p}$ is the complete quantity of the products of sector *i* demanded for each unit of the final products produced by sector *j*; the complete consumption coefficient obtained is $C^{P}={\left(I-A^{P}\right)}^{-1}-I$, thus the element $c_{ij}^{p}$ represents the number of each sector’s products or services that need to be completely consumed (i.e., the sum of direct and indirect consumption) for each unit of final products produced by sector *j*. Since $C^{P}$ only represents the sum of each sector’s direct and indirect consumption, in addition to the content included $C^{P}$, $B^{P}$ also includes the final products produced by each sector. Therefore, when calculating the embodied energy, it is more accurate to use the product of $B^{P}$ times sectors’ direct energy consumption intensity matrix as the total energy consumption coefficient.

Assuming that the direct energy consumption coefficient of sector *i* in country *P* is $e_{i}^{p}$, then the complete energy consumption of sector *i* is $\alpha_{i}^{p}=e_{i}^{p}\times B^{p}$, and the embodied energy consumption per unit of output value is $\beta_{i}^{p}$ = $\alpha_{i}^{p}$.

If the amount of China’s exports to country *P* is $ex_{i}^{CN,P}$, then:

(4) $$EX^{CN,P}=\sum_{i=1}^{m}\beta_{i}^{CN}*ex_{i}^{CN,p}$$

(5) $$EX^{CN}=\sum_{p=1}^{r}EX^{CN,P}=\sum_{p=1}^{r}\sum_{i=1}^{m}\beta_{i}^{CN}*ex_{i}^{CN,p}$$

where $EX^{CN,P}$ is the embodied energy of China’s export to country *P* and $EX^{CN}$ is the embodied energy of China’s export.

(6) $$EEX=EI{\left(I-A^{D}\right)}^{-1}EX$$

where EEX is the total amount of the embodied energy of China’s export, of which the unit is 10,000 tons of coal equivalent; EI is the direct energy consumption coefficient matrix per unit of China’s total output, a 1 × m matrix, of which the unit is 10,000 tons of coal equivalent per CNY 10,000; *I* is a unit square matrix of order m; $A^{D}$ is China’s direct consumption coefficient matrix after deducting imported intermediate products; and EX is the export matrix, an m × 1 matrix, of which the unit is CNY 10,000. Given that China’s input–output tables for 2002, 2007, and 2012 are competitive ones, this paper adopts the method of distribution in equal proportions (i.e., the proportion of imported intermediate inputs in the total intermediate use equals the proportion of imported goods in the final goods) to deduct the embodied energy of imported intermediate inputs to keep the calculation results of embodied energy of export from overestimation. Therefore, when calculating the embodied energy of China’s export in 2002, 2007, and 2012, Equation (6) is adjusted to Equation (7):

(7) $$EEX=EI_{}{\left[I-(I-M)A\right]}^{-1}EX$$

where A is China’s direct consumption coefficient matrix, an m × m matrix; and M is the import coefficient matrix, which is a diagonal square matrix of order m. According to the method of distribution in equal proportions, the element $m_{ii}$ on the diagonal line can be expressed as:

(8) $$m_{ii}=\frac{IM_{i}}{X_{i}+IM_{i}-EX_{i}}$$

where $X_{i}$ is the total output of sector *i*, of which the unit is CNY 100 million; $IM_{i}$ is the gross imports of sector *i*, of which the unit is CNY 100 million; and $EX_{i}$ is the gross exports of sector *i*, of which the unit is CNY 100 million.

The energy consumption data used in this paper are quoted from the China Energy Statistical Yearbook 2019, and the import and export data are provided by the National Bureau of Statistics of China. This paper also uses the input–output tables (China’s input–output tables are not compiled every year. Generally, input–output surveys in China are carried out in the years ending in 2 and 7 and the input–output tables for the corresponding years are compiled. Additionally, input–output extension tables are compiled in the years ending in 0 and 5. In order to reduce errors as much as possible, this paper uses the input–output table generated from actual surveys in the years ending in 2 and 7 to conduct research. The input–output table of 2018 is the first input–output table compiled in conjunction with census data in the year of China’s economic census. Therefore, this paper finally conducts research based on the input–output tables for 2002, 2007, 2012, and 2018) for 2002, 2007, 2012, and 2018. Relevant prices are adjusted by the use of the double reduction method with 2002 as the base period. According to the classification of 17 sectors in the basic input–output flow tables of the China Statistical Yearbooks (the data from China Energy Statistical Yearbooks and National Bureau of Statistics of China do not contain energy consumption by real estate, leasing and business services, and financial intermediation so the above two sectors are included into other services), this paper lists 15 sectors in the input–output tables after merging, i.e., agriculture, forestry, animal husbandry, fishery, mining, foods, beverage and tobacco, textile, wearing apparel and leather products, other manufactured products, coking, gas and oil refining, chemical products, non-metallic mineral products, metal manufacture, machinery and equipment, production and supply of electric power and heat power, construction, transport, storage and post, information transmission, computer service and software, wholesale and retail trades, lodging and catering, and other services. Regarding the merging of sectors, there are some differences between studies. For example, scholars such as Zhang et al. [20] and Xu et al. [24] listed 15 sectors in input–output tables after merging; Xu et al. [15] and Chen et al. [18] listed 17 sectors after merging; and Mi et al. [9], Chen et al. [12], Wei et al. [17], and Zhu et al. [25] listed more than 30 sectors after merging. Although these studies applied different merging methods, the final research conclusions are comparable.

## 3. Calculation Results and Analysis

### 3.1. Successful Absolute Decoupling of China’s Growing Exports in Foreign Trade from Embodied Energy of Export

The calculation results show that the embodied energy of China’s export showed the following trends from 2002 to 2018: (i) The absolute amount of embodied energy of export increased from 339 million tons of coal equivalent in 2002 to 980 million tons of coal equivalent in 2007, then grew to 1.261 billion tons of coal equivalent in 2012 before falling to 1.254 billion tons of coal equivalent in 2018, showing a changing trend from growth to decline. (ii) The proportion of embodied energy of export in China’s energy consumption also showed a trend from growth to decline, standing at 26.57% according to the calculation results of the input–output table for 2018, which is higher than the proportion in 2002 but 4.91 percentage points lower than the 31.48% in the peak period of 2007. (iii) The total value of export commodities showed a linear growth trend (fluctuating during 2008–2009 due to the global economic crisis), increasing from CNY 2.69 trillion in 2002 to CNY 11.12 trillion in 2018. 

By referring to Equation (9), a method for creating decoupling indexes proposed by Tapio [3], this paper conducts a quantitative analysis of the decoupling development trend of energy embodied in exports of trade commodities.

(9) DI=%ΔEEX/%ΔEX

where DI represents the decoupling indexes, ΔEEX is the amount of change in embodied energy of export between two adjacent periods, and %ΔEEX is the change rate of corresponding embodied energy of export; ΔEX is the amount of change in gross exports between two adjacent periods and %ΔEX is the change rate of corresponding gross exports.

**Figure 1:**
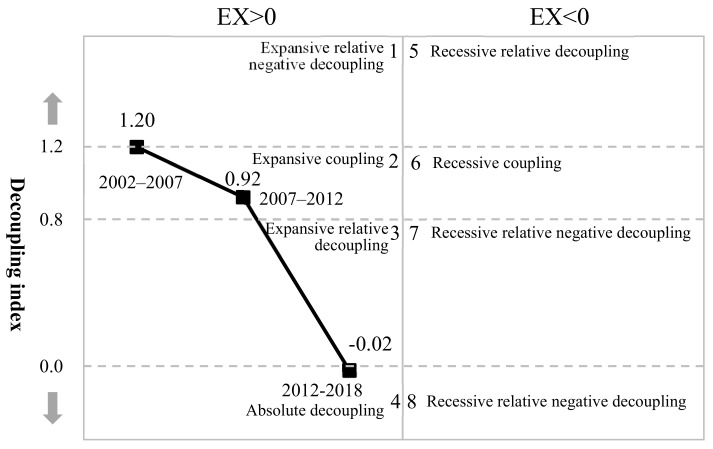
Classification of decoupling between gross exports and embodied energy of export with decoupling indexes.

According to China’s current economic development and assuming that the growth rate of gross exports is positive, when %ΔEEX > 0 but %ΔEEX < %ΔEX, it is called a period of relative decoupling; when %ΔEEX < 0, it is called a period of absolute decoupling. The calculation results show (see Figure 1 and Table 1) that the decoupling indexes in the three stages of 2002–2007, 2007–2012, and 2012–2018 correspond to different stage characteristics of decoupling, varying greatly from one stage to another: the decoupling index of 2002–2007 is 1.20, which is located in the expansive coupling zone; the decoupling index of 2007–2012 is 0.92, which is located in the expansive coupling zone; the decoupling index of 2012–2018 is −0.02, which is located in the absolute decoupling zone.

### 3.2. Analysis of the Causes of Decoupling

Structural decomposition analysis (SDA) and index decomposition analysis (IDA) are two research methods commonly used to analyze the factors influencing the changes in embodied energy of export. After analyzing both approaches, the SDA weighted average method is more perfect and precise in theory [19]. Therefore, this paper uses the SDA weighted average method to structurally decompose the changes in the embodied energy of China’s export, that is, analysis and discussion of the main reasons for the changes in the embodied energy of export from the aspects of technology effect, scale effect, and structural effect. While technology effect is reflected by the total energy consumption coefficient, the scale effect is brought into the analysis via gross exports and the structural effect is the proportional structure of export commodities. The details are as follows:

Equations (6) and (7) are further expanded into:

(10) $$EEX=EI{\left(I-A^{D}\right)}^{-1}EX=QWR$$

(11) $$EEX=EI_{}{\left[I-(I-M)A\right]}^{-1}EX=QWR$$

where Q is the matrix of total energy consumption coefficient of each sector, a 1 × m matrix, of which the element is $q_{i}$ and the unit is 10,000 tons of coal equivalent per CNY 10,000; W is the export structure matrix of each sector, an m × 1 matrix, of which the element $\omega_{i}$ is the proportion of exports of sector *i* in gross exports, i.e., $\omega_{i}=ex_{i}/R$; and R is the gross exports, i.e., $R=\sum_{i=1}^{m}ex_{i}$, of which the unit is CNY 10,000. Therefore, changes in Q are technology effect, changes in W are structural effect, and changes in R are scale effect. At the same time, the embodied energy of export of sector *i* can be decomposed into Equation (12) according to the three effects.

(12) $$eex_{i}=q_{i}*ex_{i}=q_{i}*\omega_{i}*R_{}$$

Using 0 and 1 to represent the base period and the target period, ΔEEX represents the amount of change in embodied energy of export between the two periods, namely:

(13) $$\Delta EEX=EEX_{1}-EEX_{0}=Q_{1}W_{1}R_{1}-Q_{0}W_{0}R_{0}$$

From the base period, Equation (13) can be decomposed into:

(14) $$\Delta EEX=\Delta QW_{0}R_{0}+Q_{1}\Delta WR_{0}+Q_{1}W_{1}\Delta R$$

From the target period, Equation (13) can be decomposed into:

(15) $$\Delta EEX=\Delta QW_{1}R_{1}+Q_{0}\Delta WR_{1}+Q_{0}W_{0}\Delta R$$

Since there are three variables, in addition to Equations (14) and (15), Equation (13) can be decomposed in a total of 3! = 6 ways, and therefore by calculating the weighted average of all methods of decomposition, it is possible to obtain the amount of change ΔEEX in the embodied energy of export between two periods. Let EEX(ΔQ), EEX(ΔW), and EEX(ΔR) respectively represent the influences of changes in the three factors, i.e., complete consumption coefficient, export structure, and export scale, on ΔEEX, that is, ΔEEX=EEX(ΔQ)+EEX(ΔW)+EEX(ΔR), then take EEX(ΔQ) as an example:

(16) $$EEX(\Delta Q)=\frac{1}{3}\Delta QW_{0}R_{0}+\frac{1}{6}W_{1}\Delta QR_{0}+\frac{1}{3}W_{1}R_{1}\Delta Q+\frac{1}{6}R_{1}\Delta QW_{0}$$

**Figure 2:**
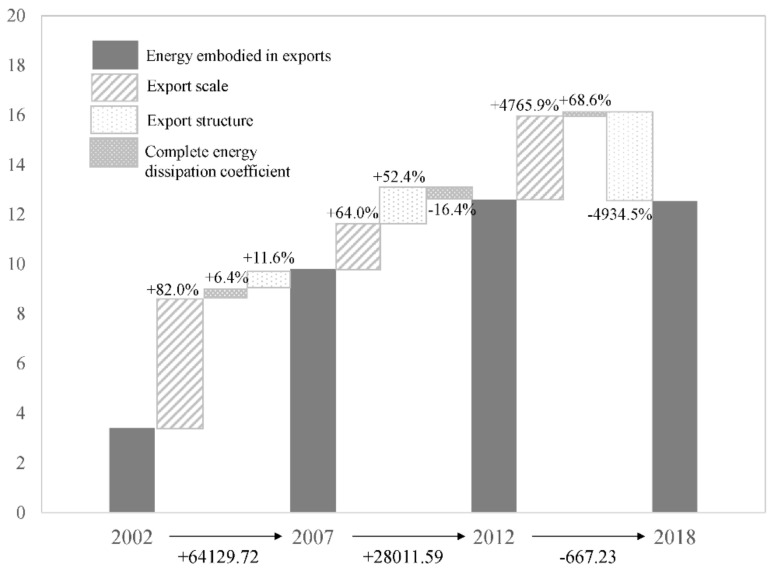
Decomposition of the contribution by changes in embodied energy of export from 2002 to 2018.

The calculation results show (see Figure 2 and Table 2) that the change in total embodied energy of export from 2002 to 2018 is a net increase, from 339 million tons of coal equivalent to 1.254 billion tons of coal equivalent, to which the rates of the contribution made by the export scale and export structure were positive, respectively 98.9% and 1.5%, while that made by total energy consumption coefficient was −0.4%. However, the large increase in the export scale offsets the negative effect of the total energy consumption coefficient, so the embodied energy of export from 2002 to 2018 showed rapid growth. It can be inferred from the results in Figure 1 that it is necessary to study the characteristics of changes in embodied energy of export by stage in order to understand the changing trend from 2002 to 2018. From 2002 to 2007, the embodied energy of export and gross exports maintained a trend of rapid growth in the same direction. The SDA analysis results indicate that the rate of the contribution made by export scale to the embodied energy of export was 82%, that by export structure was 6.4%, and the total energy consumption coefficient was −11.6%. The export scale was the main reason for the changes in the embodied energy of export. Thus, with the quick rise of export scale, the embodied energy of export also showed a trend of rapid growth. The research results provided by Pang et al. [23], Xie et al. [19], Chen et al. [18], and Liu et al. [22] also showed that the scale effect was a significant factor influencing the changes in energy embodied in exports. From 2007 to 2012, the embodied energy of export and the gross exports maintained a trend of slow growth in the same direction. The SDA analysis results showed that the rates of contribution made by the export scale and total energy consumption coefficient to the embodied energy of export were positive, respectively 64.0% and 52.4%, and that by export structure was negative, standing at 16.4%. The export scale was the main reason for the increase in the embodied energy of export. From 2012 to 2018, while the gross exports continued to rise, the total embodied energy of export declined, showing a relationship of absolute decoupling between the two. The SDA analysis results showed that the declining total energy consumption coefficient was the main reason for the decoupling, with its contribution rate standing at 4934.5%, while the export scale and export structure in the same period made a negative contribution to the decline in the embodied energy of export. The decrease in the total energy consumption coefficient is related to China’s vigorous promotion of ecological civilization and green and low-carbon development since 2012. China’s energy consumption per unit of GDP has dropped from 0.75 tons of coal equivalent per CNY 10,000 in 2012 to 0.57 tons of coal equivalent per CNY 10,000 in 2020 (calculated at comparable prices in 2012); the proportion of coal in total energy consumption has dropped significantly from 68.5% in 2012 to 56.8% in 2020 (Source: Calculated on the basis of relevant data from the database of National Bureau of Statistics of China). To further test the contribution made by the effect of the total energy consumption coefficient to decoupling, this paper uses the total energy consumption coefficient of 2012 to replace that of 2018 and works out the embodied energy of export of 2018 under the conditions of the export scale and export structure of 2018. The results show that the embodied energy of export increased to 1.62 billion tons of coal equivalent in 2018, which means that the increase in embodied energy of export contributed only by the change in total energy consumption coefficient was 369.92 million tons of coal equivalent. This is comparable to the effect of the contribution made by the changes in total energy consumption coefficient of 2018/2012 as the SDA decomposition results, i.e., 329.25 million tons of coal equivalent.

## 4. Conclusions

Based on the input–output tables for 2002, 2007, 2012, and 2018, this paper calculates the embodied energy of export in China and every sector, and uses the SDA weighted average method to analyze the reasons for the changes in embodied energy of export from 2002 to 2018. The main conclusions drawn therefrom are as follows.

(i) The embodied energy of China’s export and the total export was strongly decoupled for the first time from 2012 to 2018, signaling that China’s economic, industrial, and energy structures entered a new stage. It was also the first absolute decoupling achieved in the process of decoupling economic growth from energy consumption for the adoption of a low-carbon development path. Due to the pressure of international competition, the export sector had a relatively advanced level of efficiency and thus achieved decoupling earlier than the overall manufacturing sector and consumer sector, which was in line with economic laws and the characteristics of China’s development stage.

(ii) From 2007 to 2018, the embodied energy of export occupied a much smaller proportion of China’s total energy consumption, falling from the peak of 31.48% in 2007 to 26.57%, a drop of 4.91 percentage points. It showed that a larger share of energy consumption had taken place domestically and that the model mainly relying on export expansion to drive economic growth had begun to adjust. The conclusion of this research could also support the assertion of ‘accelerating the construction of a new development pattern with the domestic economic cycle as the main body and the domestic and international dual cycles promoting each other’ from the perspective of external exports and energy consumption.

(iii) A causal analysis of the decoupling between the embodied energy of export and export volume demonstrated that, from 2002 to 2007 and from 2007 to 2012, the embodied energy of export and total export maintained the same direction but had different growth rates. The increase in total export volume was the main reason affecting the embodied energy of export. With the rapid growth of total export volume, the embodied energy of export was also growing rapidly. From 2012 to 2018, the total embodied energy of export declined and an analysis showed that the ‘total energy consumption coefficient’, i.e., technology effect, was the primary cause of the decline. With China’s high-quality development, green transformation, and other strategic advancements, not only does the energy structure continue to transform to renewable energy, but energy efficiency also continues to improve and the decoupling trend is expected to continue and expand to a larger economic field.

## Figures and Tables

**Table 1:** Decoupling index and category from 2002 to 2018.

Period	Decoupling Index	Decoupling Category
2002–2007	1.20	Expansive relative decoupling
2007–2012	0.92	Expansive relative decoupling
2012–2018	−0.02	Absolute decoupling

Note: The decoupling categories are based on Tapio [3] and Zhang et al. [7].

**Table 2:** Contribution rate of change of embodied energy of export from 2002 to 2018.

Period	Change of Embodied Energy of Export/Million tce	Contribution Rate of Change of Embodied Energy of Export/%		
		Export Scale	Export Structure	Total Energy Consumption Coefficient
2002~2007	641.30	82.00	6.40	11.60
2007~2012	280.12	64.00	−16.40	52.40
2012~2018	−6.67	4765.90	68.60	−4934.50
2002–2018	914.75	98.90	1.50	−0.40

## Data Availability

The China Energy Statistical Yearbook 2019 is at https://data.cnki.net/yearbook/Single/N2020120303 (accessed on 21 September 2021); The National Bureau of Statistics of China is at https://data.stats.gov.cn/easyquery.htm?cn=C01 (accessed on 21 September 2021); The China’s input–output tables for 2002, 2007, 2012, and 2018 are at https://data.stats.gov.cn/ifnormal.htm?u=/files/html/quickSearch/trcc/trcc01.html&h=740 (accessed on 21 September 2021).

## References

[1] World Resources Institute (WRI) The Roads to Decoupling: 21 Countries Are Reducing Carbon Emissions While Growing GDP 2016. https://www.wri.org/insights/roads-decoupling-21-countries-are-reducing-carbon-emissions-while-growing-gdp.

[2] Ma L., Liu L. (2016). Peak forecast of Chinese energy consumption based on developed countries’ trends. Sci. Geogr. Sin..

[3] Tapio P. (2005). Towards a theory of decoupling: Degrees of decoupling in the EU and the case of road traffic in Finland between 1970 and 2001. Transp. Policy.

[4] Diakoulaki D., Mandaraka M. (2007). Decomposition analysis for assessing the progress in decoupling industrial growth from CO_2_ emissions in the EU manufacturing sector. Energy Econ..

[5] Pan J., Zhang L. (2011). Research on the regional variation of carbon productivity in China. China Ind. Econ..

[6] Sheng Y., Ou M., Liu Q. (2015). Methods of measuring decoupling of resource environment: Speed decoupling or quantity decoupling?. China Popul. Resour. Environ..

[7] Zhang C., Cai W., Yu T. (2013). Regional economic development and carbon productivity—A convergent and decoupling index analysis. China Ind. Econ..

[8] Chen Z., Chen G. (2011). An overview of energy consumption of the globalized world economy. Energy Policy.

[9] Mi Z., Meng J., Guan D., Shan Y., Song M., Wei Y., Liu Z., Hubacek K. (2017). Chinese CO_2_ emission flows have reversed since the global financial crisis. Nat. Commun..

[10] Cui L., Han J., Sun J. (2014). Study on embodied energy in international trade against globalization. J. Int. Trade.

[11] Guo C., Hu Y. (2019). Research on the cross-border transfer of embodied energy under global production division system. China Popul. Resour. Environ..

[12] Chen Y., Pan J., Xie L. (2008). Energy embodied in goods of international trade in China: Calculation and policy implications. Econ. Res. J..

[13] Qi Y., Li H., Xu M. (2008). Accounting embodied carbon in import and export in China. China Popul. Resour. Environ..

[14] Tang X., Snowden S., Hoeoek M. (2013). Analysis of energy embodied in the international trade of UK. Energy Policy.

[15] Xu P., Wang T. (2014). Analysis of embodied CO_2_ in China-UK trade. Int. Econ. Trade Res..

[16] Yang R., Long R., Yue T., Shi H. (2014). Calculation of embodied energy in Sino-USA trade: 1997–2011. Energy Policy.

[17] Wei T., Peng S. (2017). Research on embodied energy in China-USA trade with MRIO model. Soft Sci..

[18] Chen W., Li Q. (2014). The energy consumption by China’s foreign trade: An analysis based on non-competitive input-output method. J. World Econ..

[19] Xie J., Jiang P. (2014). Embodied energy in international trade of China: Calculation and decomposition. China Econ. Q..

[20] Zhang H., Jiang Y. (2016). Embodied energy calculation of China’s foreign trade based on the improved method. China Popul. Resour. Environ..

[21] Gu A., He J., Zhou L., Yao L., Liu B. (2010). Analysis of embodied energy and transfer emissions of China’s import and export trade. J. Tsinghua Univ. (Sci. Technol.).

[22] Liu X., Huang X. (2015). Accounting embodied energy and environmental analysis in China’s import and export: Based on the correction method of input-output. Stat. Inf. Forum.

[23] Pang J., Shi Y., Yan Y., Huang S. (2012). China’s energy consumption embodied in exports and the decomposition analysis of its influencing factors. Inq. Into Econ. Issues.

[24] Xu D., Dong B. (2012). Analysis of deficit of ecology and eco-friendly trade—Based on energy embodied in China. East China Econ. Manag..

[25] Zhu Q. (2011). Research on the energy consumption by China’s exports. Stat. Res..

